# Age-related declines in a two-day reference memory task are associated with changes in NMDA receptor subunits in mice

**DOI:** 10.1186/1471-2202-8-43

**Published:** 2007-06-22

**Authors:** Kathy R Magnusson, Brandi Scruggs, Xue Zhao, Rebecca Hammersmark

**Affiliations:** 1 Department of Biomedical Sciences, College of Veterinary Medicine, Oregon State University, Corvallis, OR, 97331, USA; 2 Department of Biological Sciences, University of Idaho, College of Science, Moscow, ID, 83844, USA; 3 Behavioral testing performed at: Department of Biomedical Sciences, College of Veterinary Medicine and Biomedical Sciences, Program in Molecular, Cellular, and Integrative Neurosciences and Department of Psychology, Colorado State University, Fort Collins, CO, 80523, USA; 4 Department of Psychology, Worcester State College, Worcester, MA 01602-2597, USA

## Abstract

**Background:**

C57BL/6 mice show a relationship during aging between NMDA receptor expression and spatial reference memory performance in a 12-day task. The present study was designed to determine if age-related deficits could be detected with a shorter testing protocol and whether these deficits showed a relationship with NMDA receptors. Mice were trained in a reference memory task for two days in a Morris water maze. Cued testing was performed either after or prior to reference memory testing. Crude synaptosomes were prepared from prefrontal/frontal cortex and hippocampus of the mice that underwent reference memory testing first. NMDA receptor subunit and syntaxin proteins were analyzed with Western blotting.

**Results:**

Young mice showed significant improvement in probe and place learning when reference memory testing was done prior to cued testing. A significant decrease in performance was seen between 3 and 26 months of age with the two-day reference task, regardless of whether cued testing was performed before or after reference memory testing. There was a significant decline in the protein expression of the ε2 and ζ1 subunits of the NMDA receptor and syntaxin in prefrontal/frontal cortex. The subunit changes showed a significant correlation with both place and probe trial performance.

**Conclusion:**

The presence of an age-related decline in performance of the reference memory task regardless of when the cued trials were performed suggests that the deficits were due to factors that were unique to the spatial reference memory task. These results also suggest that declines in specific NMDA receptor subunits in the synaptic pool of prefrontal/frontal brain regions contributed to these age-related problems with performing a spatial reference memory task.

## Background

Memory is one of the earliest of the cognitive functions to show declines during the aging process [1]. Memory deficits associated with aging are seen in humans and non-human primates (see reviews [2,3]), dogs [4] and rodents [5-8]. One type of memory that is important for how individuals cope with their environment is spatial memory. Humans show 30% to 80% drops in performance in spatial memory tasks as they age [9-14]. Mice and rats also exhibit deficits in spatial memory performance during aging [6-8,15-19].

**Figure 1:**
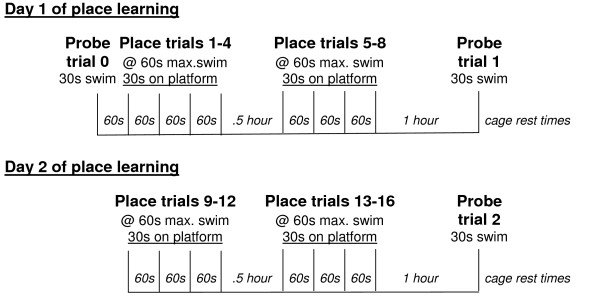
**Diagram of the protocol for reference memory testing over a two day period, including both place learning and probe trials**. s, seconds; max., maximum.

Aged C57BL/6 mice show spatial reference memory problems when tested over 12 days in the Morris water maze [17-19]. We were interested in adopting a task that would show age-related deficits in fewer days in order to test drug interventions with the use of osmotic pumps (Durect Corp., Cupertino, CA). The smallest pump available can deliver drug for 3–14 days, but, in addition to the time necessary for behavioral testing, time is also needed for recovery from surgery and pretraining. Berry and coworkers developed a one-day spatial memory task for rats, in which young rats show good improvement in performance 8 trials in one day [20]. Our initial attempts to use this one-day task with mice showed that young mice could not show a significant improvement within 8 trials in one day, but could with two days of testing (unpublished observation). The present study was designed to determine whether we could detect significant differences in performance between young and old mice in a spatial reference memory task with a two-day testing protocol (Figure 1).

The N-methyl-D-aspartate (NMDA) receptor, a subtype of glutamate receptors, is particularly important in learning and memory functions [21,22]. NMDA antagonists inhibit memory performance [23-26] and block the initiation of long-term potentiation in the hippocampus [26-28] and neocortex [29]. These studies suggest that detrimental changes to the NMDA receptor during the aging process should impact negatively on memory functions.

Aging animals do exhibit declines in NMDA receptor binding densities and functions, including memory-related functions. NMDA-stimulated release of transmitters is decreased with increasing age [30,31]. Long-term potentiation is also altered in aged rodents [32,33]. Age-related declines in binding of glutamate and [(±)-2-carboxypiperazin-4-yl] propyl-1-phosphonic acid (CPP) to NMDA binding sites have been reported in mice, rats, dogs, and monkeys [34-39]. Humans also exhibit declines with increased age in binding of [^3^H]MK801 to the NMDA receptor complex in the frontal cortex [40]. Changes in NMDA binding sites during aging have been correlated with poor performance in reference memory tasks, such as the Morris water maze, in both prefrontal/frontal cortical regions [17] and in the hippocampus [17,37].

Functional subunits of the NMDA receptor complex have been cloned for rats [41-43] and mice [44-47]. The zeta1 (ζ1; rat NR1) subunit has the same distribution as NMDA-displaceable [^3^H]glutamate binding [41,46,48]. The four members of the epsilon family of subunits, ε1–4 (rat NR2A-D), in the mouse [44-46] each provide different agonist/antagonist affinities to ζ1/ε heteromeric receptors [45,47]. These ε subunits also produce different gating behaviors, responses to Mg^++^, and I/V curves [42,43].

There is a difference in the effects of aging on different subunits of the NMDA receptor. The density of mRNA expression for the ε2 subunit declines with increasing age in the cerebral cortex and dentate gyrus of male C57BL/6 mice [38]. These changes in ε2 mRNA expression correlate significantly with age-related changes in binding of agonist to NMDA sites both across cortical and hippocampal regions [38] and within prefrontal/frontal cortical regions [18]. There is also an overall decrease in mRNA expression of the ζ1 subunit across cortical and hippocampal regions, but to a lesser degree than seen with the ε2 subunit [38]. Although age-associated changes in ε2 mRNA in the prefrontal/frontal cortex correlate with changes in NMDA receptor binding and the binding correlates with spatial reference memory problems [18], there has not been a direct relationship seen between the mRNA expression of the NMDA receptor and memory performance [18]. C57BL/6 mice show significant decreases with increasing age in the protein expression of ε2 and ζ1 subunits in homogenates from the whole cerebral cortex [49]. Significant declines during aging in protein expression in hippocampal homogenates are also seen in the ζ1 and ε2 subunits in both mice [49] and Fisher 344 rats [50,51]. Homogenates of only prefrontal/frontal cortex show a significant age-related decrease in the protein expression of the ε2 subunit alone [52]. Given the differences in pharmacological and electrophysiological properties that different combinations of subunits produce [42,43,45,47], it is important to determine if any of these subunit changes relate to memory performance declines during aging. It seems likely that the relationship between the receptor subunits and memory ability would be strongest with the protein expressed in the synaptic pool since this pool would be enriched in receptors that are functioning in neurotransmission. We hypothesized that there would be a relationship between the ε2 and ζ1 subunit expression in the synaptic pool and reference memory performance. The ε1 subunit does not show significant changes in mRNA during aging and less change in protein expression than ε2 and ζ1 [18,38,49], but ratios of high mRNA expression of ε1 in the face of lower expression of ε2 or ζ1 do show a relationship to reference memory performance [18]. It was expected that the protein expression of the ε1 subunit in the synaptic pool might not change significantly with age, but might show a relationship with reference memory performance.

The present study was designed to determine whether there was a relationship between NMDA receptor subunit expression within crude synaptosomes and spatial reference memory ability in a two-day task. The main study was done with the reference task performed prior to the cued task because this most closely resembled the longer reference memory tasks that have been used previously in this lab. Additional animals from the same shipment performed the cued task first in case some previous training in the mechanics of the water maze task (e.g., getting on the platform, recognizing the platform as a means to escape) was necessary to see significant learning within 2 days in the reference memory task. A crude synaptosome preparation was used because it should be enriched in NMDA receptors that are in the synaptic pool [53]. The analysis of the protein expression of syntaxin was intended to be used as a loading control for the Western blot, but it showed significant changes during aging in the prefrontal/frontal cortex and could not be used for this purpose.

## Results

### Behavioral testing – place training trials followed by cued trials

#### Place trials

**Figure 2:**
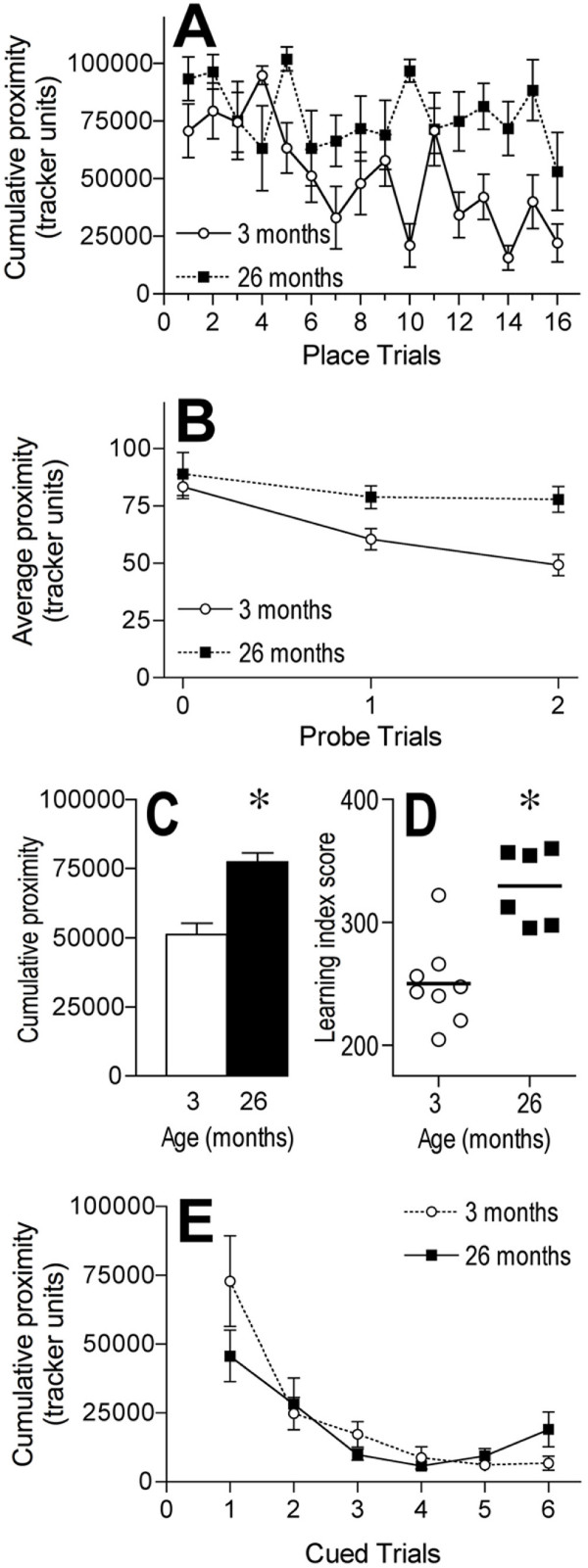
**Effects of age on reference memory and cued tasks with cued trials conducted last**. A and C: Graphs showing the performance, measured as cumulative proximity in tracker system units, of 3 and 26 month old mice within individual place learning trials (A) and averaged across all place learning trials (C). B: Graph showing the performance, measured as average proximity in tracker system units, of 3 and 26 month old mice within individual probe trials. D: Individual learning index scores with the horizontal bar indicating the mean for each age group. E: Graph showing the performance, measured as cumulative proximity in tracker system units, of 3 and 26 month old mice within cued trials in the water maze. * *p *< .05 for difference from 3 month old mice (analysis of variance and Fisher's protected least significant difference post-hoc analysis). *n *= 8 for 3 month olds and *n *= 6 for 26 month old mice. Error bars represent *SEM*.

There was a significant overall effect of age, *F *(1, 12) = 22.785, *p *= .0005, and trial, *F *(15, 180) = 3.135, *p *= .0001, on performance in place learning trials (Figure 2A, C). There was also a significant interaction between age and trial, *F *(15, 180) = 2.408, *p *= .0033, on place learning performance. The 26 months olds had significantly greater cumulative proximity measurements than 3-month-old mice when performance was averaged across all place trials (Figure 2C). The 3 month olds showed a significant reduction in cumulative proximity between the first place trial of Day 1 and last trial of Day 2 (trial 16; *p *= .0041), but not between the first and eighth (last trial of Day 1) place trials (*p *= .2225; Figure 2A). The 26-month-old mice showed only a near significant decrease (*p *= .0641) in cumulative proximity from the first to the last place trial and no significant change between place trials 1 and 8 (Day 1; *p *= .2313; Figure 2A). There was no significant main effect of age, *F *(1, 12) = 3.396, *p *= .0902, on cumulative proximity measurements across place learning trials 1 through 8 (Day 1; Figure 2A).

#### Probe trials

Probe trials 0 and 1 were performed on Day 1 and Probe trial 2 on Day 2 (Figure 1). There was a significant overall effect of age, *F *(1, 12) = 19.006, *p *= .0009, and trial, *F *(2, 24) = 7.154, *p *= .0037, on performance in probe trials (Figure 2B). The 26 months olds (82 ± 3 (SEM) tracker units) had significantly higher average proximity measurements than 3-month-old mice (64 ± 3 tracker units) when performance was averaged across all probe trials. The old mice had significantly greater average proximity measurements than young on probe trials 1 and 2 (*p *= .02 and .0019, respectively), but there was no difference between the two age groups in performance on the initial probe trial (*p *= .5915; probe trial 0; Figure 2B). The 3 month olds showed a significant reduction in average proximity between probe trials 0 and 1 (*p *= .0053) and probe trials 0 and 2 (*p *= .0002; Figure 2B). The 26-month-old mice showed no significant change in average proximity measurements between probe trials 0 and 1 (*p *= .3654) or probe trials 0 and 2 (*p *= .3381; Figure 2B). There was a significant main effect of age, *F *(1, 12) = 8.673, *p *= .0123, on average proximity across probe trials 0 and 1 (Figure 2B). Learning index scores were derived for each animal from the performance across the probe trials [54]. There was a significant main effect of age on learning index scores *F *(1, 12) = 19.413, *p *= .0009 with old mice having higher scores than young (Figure 2D).

#### Cued trials

There was no significant overall effect of age, *F *(1, 12) = .393, *p *= .54, but there was a significant overall effect of trial, *F *(5, 60) = 15.962, *p *< .0001, on performance in cued trials when they occurred after the place training trials (Figure 2E). Both the young and old mice exhibited a significant reduction (*p *= .0014 and .0396, respectively) in cumulative proximity measurements between the first and last cued trials (Figure 2E). There were no significant differences between the age groups in any of the individual cued trials (*p *range = .0689 – .75; Figure 2E).

### Behavioral testing – cued trials prior to place training trials

#### Cued trials

**Figure 3:**
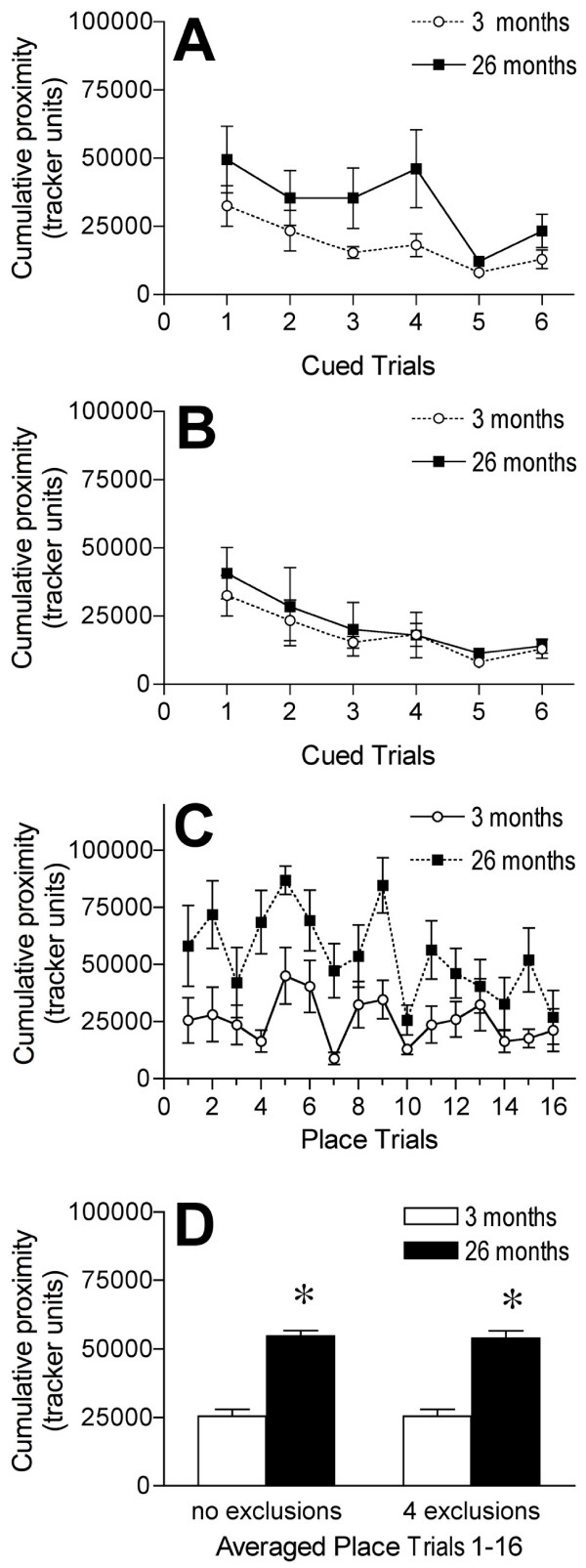
**Effects of age on cued and reference memory tasks with cued trials conducted first**. A and B: Graphs showing the performance, measured as cumulative proximity in tracker system units, of 3 and 26 month old mice within cued trials in the water maze with all animals included in analysis (A) and with 4 aged mice excluded for poorer performance on cued trials than 3 standard deviations from the mean performance in the young (B). C: Graph showing the performance, measured as cumulative proximity in tracker system units, of 3 and 26 month old mice within individual place learning trials with 4 aged mice excluded. D: Graphs showing the performance, measured as cumulative proximity in tracker system units, of 3 and 26 month old mice averaged across all place learning trials. Results are for both when all mice were included (no exclusion) and when 4 old mice were excluded (4 exclusions) from analysis. * *p *< .05 for difference from 3 month old mice (analysis of variance and Fisher's protected least significant difference post-hoc analysis). A and D (no exclusions): *n *= 8 for 3 month olds and *n *= 10 for 26 month old mice. B, C, and D (4 exclusions): *n *= 8 for 3 month olds and *n *= 6 for 26 month old mice. Error bars represent *SEM*.

There was a significant overall effect of age, *F *(1, 16) = 5.331, *p *= .0346, and there was a significant overall effect of trial, *F *(5, 80) = 3.714, *p *= .0045, on performance in cued trials when they occurred prior to the place training trials (Figure 3A). The older mice had higher cumulative proximity measurements than the young overall in the cued trials (Figure 3A). The performance averaged across cued trials 2–6 for four of the old mice was greater than 3 standard deviations from the mean of the performance of young mouse group. When these four mice were excluded from the analysis, there was no significant overall effect of age, *F *(1, 12) = .722, *p *= .41, but there was still a significant overall effect of trial, *F *(5, 60) = 4.437, *p *= .0017, on performance in cued trials that were performed prior to the place training trials (Figure 3B).

#### Place and probe trials following the cued trials

There was a significant main effect of aging on performance in place trials following the cued trials, both when the four old mice that did poorly in the cued trials were excluded, *F *(1, 16) = 57.361, *p *< .0001 (Figure 3C, D), and when they were included in the analysis, *F *(1, 16) = 82.685, *p *< .0001, (Figure 3D). The 26 month old mice had higher cumulative proximity measurements than the 3 month olds overall in the place trials, both when all mice were included (Figure 3D) and when the four were excluded (Figure 3C, D). The 3 month old mice did not show a significant improvement in cumulative proximity between place trials 1 and 16 (Figure 3C). There was a significant main effect of aging on performance in probe trials when place training followed the cued trials, both when the four old mice that did poorly in the cued trials were excluded, *F *(1, 12) = 14.35, *p *= .0026, and when they were included in the analysis, *F *(1, 16) = 22.762, *p *= .0002, (not shown). The old mice had higher average proximity measurements overall in the probe trials than the younger mice (not shown).

### Protein expression of NMDA receptor subunits in crude synaptosomes

**Figure 4:**
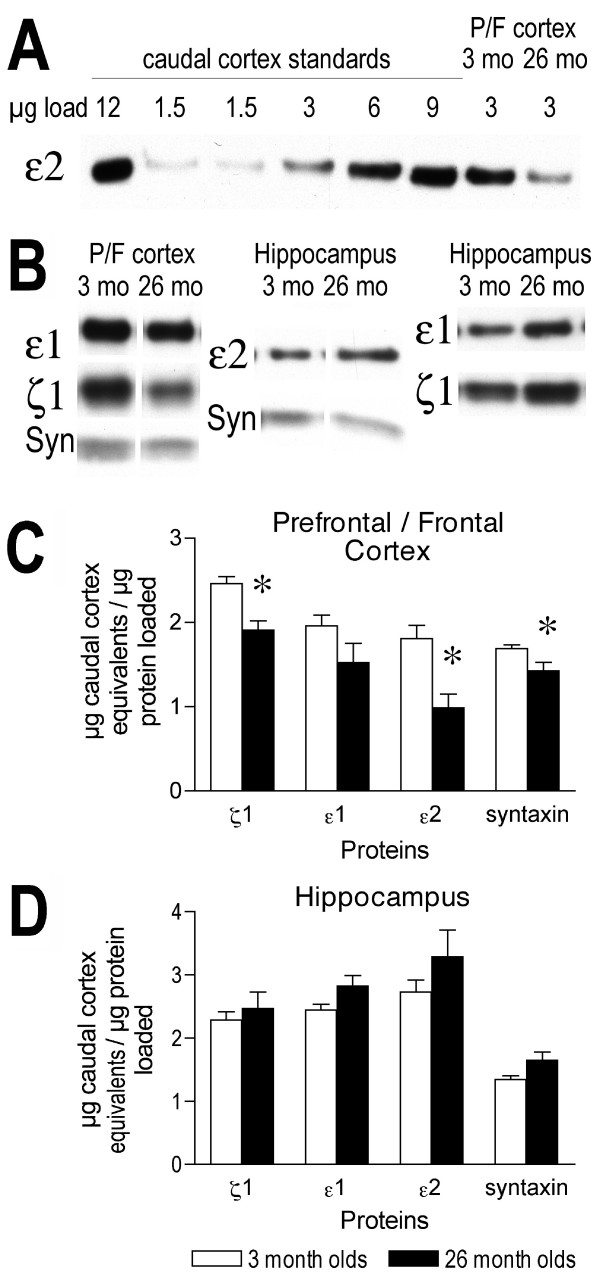
**Effects of age on protein expression of NMDA receptor subunits in crude synaptosomes**. A: Representative bands from crude synaptosomes prepared from cortex and labelled with an antibody specific for the ε2 subunit. To the right are bands from different μg loads of caudal cortex used to obtain a standard curve. Five different μg amounts of caudal cortex were loaded as standards for each blot. To the left are representative bands from the prefrontal/frontal (P/F) cortex of a 3 (3 mo) and 26 (26 mo) month old mouse. B: Representative bands for all other proteins analyzed in crude synaptosomes from prefrontal/frontal cortex and hippocampus. Columns show labelling of different proteins from the same animal and well. Each well was loaded with 3 μg of crude synaptosomes. C, D: Graphs of protein expression of the ζ1, ε1, and ε2 subunits of the NMDA receptor and syntaxin in 3 and 26 month old mice, expressed as μg caudal cortex equivalents/μg protein loaded, in prefrontal/frontal cortex (C) and hippocampus (D). * *p *< .05 for difference from 3 month old mice (analysis of variance and Fisher's protected least significant difference post-hoc analysis). *n *= 8 for 3 month olds and *n *= 6 for 26 month old mice for prefrontal/frontal cortex. n = 6 for both ages for hippocampus. Error bars represent *SEM*.

Protein expression was assessed in the mice that underwent place training prior to cued training. There was a significant decrease in caudal cortex equivalents per μg of protein loaded for the ζ1 and ε2 subunits of the NMDA receptor (*p *= .0014 and .004, respectively) in crude synaptosomes from the prefrontal/frontal cortex of 26-month-old mice as compared to young (Figure 4A–C). There was no significant effect of age on caudal cortex equivalents per μg of protein loaded for the ε1 (*p *= .10) subunit of the NMDA receptor in the prefrontal/frontal cortex (Figure 4B, C). Syntaxin also showed a significant decrease in expression between 3 and 26 months of age in the prefrontal/frontal cortex (*p *= .0221; Figure 4B, C). There was no significant effect of age on caudal cortex equivalents per μg of protein loaded for the ζ1 (*p *= .5441), ε1 (*p *= .0715), or ε2 (*p *= .2568) subunits of the NMDA receptor in the hippocampus (Figure 4B, D). Syntaxin showed a near-significant increase in expression between 3 and 26 months of age in the hippocampus (*p *= .0603; Figure 4D).

### Correlations between learning performance and protein expression of NMDA receptor subunits

**Figure 5:**
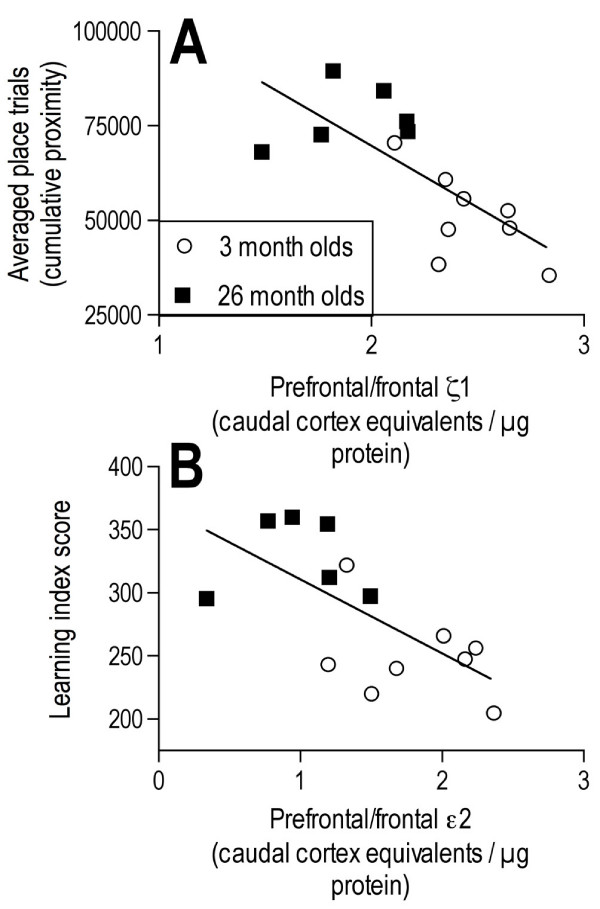
**Relationships between reference memory performance and protein expression of NMDA receptor subunits**. A, B: Correlation graphs of ζ1 (A) and ε2 (B) expression in the prefrontal/frontal cortex versus averaged place (A) or learning index score (B) derived from probe trial performance in a two-day reference memory task. Correlation coefficients are presented in Table 1. Proximity measurements are given in tracker system units.

There were significant negative correlations for both the overall cumulative proximity measurements in the place trials and the learning index scores derived from the probe trials and subunit equivalents/μg protein for the ζ1 and ε2 subunits in crude synaptosomes from the prefrontal/frontal cortex when all animals were considered (Table 1 and Figure 5). The negative correlation coefficient indicated that high levels of protein expression were associated with low proximity measurements or learning index scores (i.e., good performance in the water maze). There were no significant correlations found for protein expression of the ε1 subunit or syntaxin in the prefrontal/frontal cortex and performance in either the place or probe trials across the ages (Table 1). None of the subunits nor syntaxin expression levels in the hippocampus showed a significant correlation with place or probe trial performance across the ages (Table 1). There was a near significant correlation between the protein expression of the ε2 subunit in the hippocampus of young alone (R = -757, p = .086) and old alone (R = -77, p = .077) and the learning index score. There was a significant correlation between syntaxin expression in the hippocampus of the old group alone and the learning index score (R = -.832, p = .038).

**Table 1:** Pearson correlation coefficients (*R*) for protein expression of NMDA receptor subunits in crude synaptosomes and learning performance.

	Place trial average		Learning index score	
	
Proteins	Prefrontal/Frontal Cortex	Hippocampus	Prefrontal/Frontal Cortex	Hippocampus
ζ1	-.728 **	+.188	-.658 **	+.017
ε1	-.286	+.509	-.334	+.484
ε2	-.594 *	+.306	-.664 **	+.0003
syntaxin	-.298	+.442	-.440	+.202

* *p *< .05; ** *p *< .01; Correlations include individual mice from both age groups. N = 14 (8 young & 6 old) for Prefrontal/frontal cortex; N = 12 (6 young & 6 old) for Hippocampus.

## Discussion

This study reports the detection of age-related changes in spatial reference memory performance in C57BL/6 male mice with the use of a two-day reference memory task and changes in the protein expression of NMDA receptors within crude synaptosomes during aging. A significant decline in spatial reference learning ability in the old mice as compared to the young was detected with a two-day task, regardless of whether the cued trials were performed before or after the place training. The young mice required two days in order to show significant improvement in the place-learning task. There were significant decreases with increased age in the expression of the ζ1 and ε2 subunits of the NMDA receptor in crude synaptosomes that were prepared from prefrontal/frontal cortex and these changes appeared to be related to declines in performance ability in the spatial reference memory task.

Previous work in our laboratory utilized a reference memory task in the Morris water maze that involved 12 days of testing in order to characterize learning abilities in different ages of C57BL/6 mice [17-19]. Many other studies showing memory declines during aging in mice report the use of between 3 and 12 days to assess reference memory ability [55-61]. Frick and colleagues have used a one-day task with 12 place trials to show significant declines in memory performance between young (7 months old) and middle-aged (18 months of age) male and female C57BL/6 mice [62]. It should be noted, however, that Calhoun and co-workers found no effect of aging on reference memory performance in male C57BL/6 mice performing a reference memory task in the water maze for 5 days [63]. The present study demonstrated that declines in spatial memory ability in male C57BL/6 mice between 3 and 26 months of age could be detected in both place learning trials and repeated probe trials with a 2-day protocol of place training. These deficits in the reference memory task were seen in animals that showed no age-related differences in control cued testing, thus reducing the possibility that the performances in the reference memory task were due to problems with vision, motor ability or motivation. The performance of the aged animals in the 2-day task that was performed before the cued task showed tendencies in the middle to late trials to plateau at a level higher than the young performance or to show a worsening of performance. This pattern has been seen with C57BL/6 mice in a 12-day reference memory task [17-19] and in other studies using multiple training days for mice [55-57,59,61] and age-impaired rats [54]. Thus, this two-day task appears to be as sensitive to reference memory declines as tasks involving more days of training.

The young mice in the present study that underwent place training first showed a significant improvement in performance in probe trial performance at the end of day one as compared to the naïve probe trials, but required two days to show significant improvement in the place learning trials. The significant difference in performance between young and old mice was seen in the probe trials both during day 1 and across both days, but the age difference in place learning performance could only be detected across 2 days of training. This suggests that two days is necessary to detect the greatest differences in spatial memory ability between young and old C57BL/6 mice with a protocol of 8 place trials per day. Young female and male rats are able to show significant learning within eight place learning trials in a single day with the use of a similar protocol [20,64,65]. Our laboratory and others have also seen this necessity for using more trials for mice than rats in order to obtain significant improvement in performance in a working memory task [19,66-68]. These studies suggest that mice need more rehearsal than rats do in order to perform both reference and working memory tasks. Mice have also been shown to use different strategies to perform water maze tasks [69].

Some aged mice showed deficits in performance of the cued trials as compared to the young when the cued trials were performed first, but these mice improved in performance by cued trial 5. The majority of aged mice (6 out of 10) that were tested in the cued trials first showed similar performances to the young mice throughout the cued trials. In previous studies, both young and old mice have also been excluded from analysis after showing poor performance in cued trials that followed place training [19]. This suggests that there are some mice that can't perform well in multiple tasks in the water maze. Regardless of whether the cued trials were conducted after or prior to the place training trials, those aged mice that showed no problems in the cued task exhibited significant deficits in the place and probe trials. This suggests that the aged mice do have problems in performing the spatial reference memory task and it is not simply a problem with learning to acquire the platform, motivation, or sensory or motor abilities. What cannot be ruled out is a problem with acquiring the specific rules for the hidden platform task.

There was a significant decline between young and old mice in the protein expression of the ζ1 and ε2 subunits in crude synaptosomes from the prefrontal/frontal cortex in the present study. There was a 21% decrease in protein expression of the ζ1 subunit between 3 and 26 months of age in synaptosomes from prefrontal/frontal cortex in this study, but the protein expression of the ζ1 subunit showed a non-significant trend to decrease from 1.5 months to 25 months of age in homogenates from the caudal portion of prefrontal/frontal cortex in a previous study [52]. The protein expression of the ε2 subunit also showed a greater decrease with increased age in synaptosomes from whole prefrontal/frontal cortex (47%) than previously reported in homogenates from the caudal portion of the prefrontal/frontal cortex (17%) [52]. These results suggest that the synaptic pool of receptors is more susceptible to the effects of aging than the whole population of receptors within the neuron in the prefrontal/frontal cortex. This may reflect an increased turnover rate in the subunits either before or after they are inserted into the synaptic pool. These same subunits showed significant declines (19% decrease for ζ1 and 21% decrease for ε2) between young and old mice in homogenates of the whole cerebral cortex from the same strain of mice [49]. The ε1 subunit showed a significant decline between 10 and 30 months of age in homogenates from whole cerebral cortex [49], but those ages and extra cortical regions were not included in the present study.

The crude synaptosomes prepared from the hippocampus showed a trend for an increase in protein expression of the NMDA subunits examined. A similar pattern is seen in Wistar rat homogenates of hippocampus in which NR1 subunit proteins showed a significant increase and NR2A and NR2B subunit proteins showed a trend for an increase [70]. This differs, however, from the results from homogenates prepared from the hippocampi of C57BL/6 mice in which the ζ1 subunit showed a significant decrease in expression between 3 and 30 months of age [49]. These differences in the effects of aging on NMDA receptor expression in the synaptic pool versus the whole tissue are similar to those found in Fisher 344 rats, in which the basal surface expression of NMDA subunit proteins does not change between adult ages [71], but expression of both NR1 (ζ1) and NR2B (ε2) expression is decreased in homogenates of hippocampus from the same strain of rats [50,51]. This may represent maintenance of the synaptic pool of NMDA receptor subunits within the hippocampus during aging, but a reduction in the pool being produced. This could happen if the turnover rate at the synapse was reduced. There is also the possibility that the mice in the present study were influenced by the behavioral experience, although the rats in both of the studies mentioned above were naïve to behavioral training [50,51]. Regardless of the cause, there is a very different effect of aging on the NMDA subunit expression patterns between the prefrontal/frontal cortex and the hippocampus in C57BL/6 mice. It should be noted that Long-Evans rats do not show significant effects of aging on NR1 protein expression either by Western blotting of homogenates or immunofluorescence on tissue sections [72], so there appear to be some strain and species differences in the effects of aging on NMDA receptor expression in the hippocampus.

The protein expression of the ζ1 and ε2 subunits in the synaptosomal fraction of the prefrontal/frontal cortex correlated negatively with the proximity measurements in the place and the learning index score derived from the probe trials. Since high proximity measurements and scores indicate poor performance, the lower expression of ζ1 and ε2 in the old animals was associated with poorer performance, i.e., a positive correlation between protein and learning ability. This suggests that the changes during aging in these subunits of the NMDA receptor within the prefrontal/frontal cortex contribute to the memory problems seen in the older mice. In support of this, the administration of the ε2 subunit antagonist ifenprodil into orbital regions of the prefrontal cortex of young mice inhibited learning in the same task, suggesting that ε2 subunits in that region are involved in performing this spatial reference memory task [73].

The prefrontal cortex has been shown in both human and animal studies to be important in applying different strategies to the handling of memories that have spatial and temporal properties, in retrieval of the sources of information, and in adjusting to changing environmental conditions (reviewed in [3]). Lesioning studies in rats show that orbital and medial prefrontal regions contribute to performance in spatial reference memory tasks in the Morris water maze [74,75]. The investigators provide arguments that this is due to a deficit in organization of the behaviours necessary to perform the task or problems with handling the spatial memory temporarily, as opposed to specific spatial guidance problems [74]. Insular cortex injections of tetrodotoxin also impair the retention of platform position in the water maze [76]. More recent studies do not see an affect of lesions of the medial prefrontal cortex on performance of the spatial reference memory task in the water maze [77], but the tissue dissections in this study were not limited to medial prefrontal regions. They also included orbital and insular prefrontal regions, as well as some motor and somatosensory cortices. The literature thus provides evidence that some prefrontal regions play a role in the performance of spatial reference memory tasks. The current study suggests that declines in specific NMDA receptor subunits in the prefrontal/frontal cortex contribute to declines in performing the task by aged mice. The specific components of the performance that are affected will have to be determined.

The age-related declines seen in spatial reference memory performance of C57BL/6 mice in a 12-day protocol show a relationship with decreases in binding of the transmitter glutamate to the NMDA receptor in the prefrontal/frontal cortex and hippocampus [17,18] and to the ratio of ε1/ζ1 and ε1/ε2 subunit mRNAs within subregions of the hippocampus [18]. Changes in NR2B protein expression in homogenates from the hippocampus also show a correlation with spatial reference memory in aged Fisher 344 rats [78]. NR1 levels in the CA3 region correlate with water maze performance during aging in Long-Evans rats [72]. There was the suggestion of a relationship between the ε2 subunit expression in the hippocampus within each age group and reference memory ability, but it did not reach significance. There is a difference in how aging affects NMDA receptor binding and subunit mRNA expression between the dorsal and intermediate hippocampus in C57BL/6 mice [79], so using the whole hippocampus in this study may have diluted out some effects.

There was a significant decline in the protein expression of syntaxin, a presynaptic terminal membrane protein [80,81], in the prefrontal/frontal cortex between 3 and 26 months of age in the C57BL/6 mice in this study. For this reason, syntaxin was not used to correct for loading differences in the present study. The fact that there were differences in the effects of aging on the different NMDA receptor subunit and syntaxin suggest that the effects were not due to different amounts of protein loaded. Although we were only able to control for loading differences in this study by loading known amounts of protein, the presence of the standards on every gel do provide control for variability between gels and transfers. There was no significant effect of aging on syntaxin in the hippocampus, however, to be consistent in the reporting, the hippocampal subunits were also not normalized to syntaxin. The present results are in agreement with studies on Wistar rats that also show no declines in the protein expression of syntaxin in the hippocampus [82], but differ from results of whole cerebral cortex from Wistar rats in which syntaxin showed no aging change [83]. There was evidence though in this study that syntaxin expression within the hippocampus of the old mice may contribute to the reference memory deficits seen in the aged animals.

## Conclusion

Age-associated deficits in spatial reference memory were detected in C57BL/6 mice with the use of a two-day Morris water maze task. The most significant age-related declines in NMDA receptor subunit expression in these mice involved the ε2 subunit within the synaptic pool of the prefrontal/frontal cortex. The ζ1 subunit also showed a significant decrease during aging in this same subfraction of prefrontal/frontal cortical tissue. Lower expression of both the ζ1 and ε2 subunits across the ages was associated with poorer performance. These results suggest that changes in the synaptic pool of NMDA receptors containing ζ1 and ε2 subunits within the prefrontal/frontal cortex contributed to age-related declines in performance of a spatial reference memory task.

## Methods

### Subjects

Thirty-two, male C57BL/6JNia mice (National Institute on Aging, Bethesda, MD) representing 2 different age groups (3 and 26 months of age) were *ad libitum*-fed and housed under 12/12 hour light/dark conditions for 7 days prior to and during behavioural testing. All animal procedures were approved by the Institutional Animal Care and Use Committee at Colorado State University and conformed to NIH guidelines.

### Behavioral testing

#### Apparatus

A 4 foot diameter metal tank was filled with water (~18–20°C), made opaque with non-toxic paint, to 1 cm above the level of the platform. The spatial clues consisted of posters and geometric figures on the walls of the room and tank and the person timing the trials. There were platform positions in the center of each quadrant and 3 positions that each differed from all other positions in their distance from the wall. One of four, approximately equidistant, entry points were randomly assigned for each trial. Trials were videotaped with a Panasonic color CCD camera and VCR and path tracings were captured and analyzed with the use of a Poly-Track Video Tracking System (San Diego Instruments) and a Zenith computer. Pretraining occurred during the 2 days prior to place training and consisted of each animal swimming for 60 seconds in the tank without the platform and then being trained to remain on a platform located in the center of the tank for 30 seconds each day. One group of mice underwent place training to assess reference memory first, followed by control cued trials to assess sensory and motor ability and motivation. This was similar to the protocol that we have used previously with longer-term reference memory tasks [18,19]. A separate group of mice from the same shipment experienced cued testing prior to place training.

#### Place training trials

A two-day task for reference memory was adapted for mice (see Figure 1) from a one-day task developed by Gallagher and co-workers [20]. Preliminary experiments showed that the mice were unable to show significant learning in one day, but could with two days of training (unpublished observation). In addition, the aged mice were not able to perform 8 training trials in a row without showing signs of fatigue or stress, so 2 blocks of 4 trials with an inter-block interval of .5 hours was used. On day 1, mice underwent one naïve probe trial, one block of 4 place training trials with a 60 second intertrial interval in a cage between each trial, 30 minutes of cage rest, another block of 4 place training trials with a 60 second intertrial interval between each trial, 1 hour of cage rest, and a final probe trial (see Figure 1). Day 2 was the same except that there was no probe trial at the beginning of training (Figure 1). The platform remained in the same quadrant (NE) for all the place platform trials. During the place trials, the mice were placed in the tank facing the wall, and were allowed to search for the platform for 60 seconds. If they had not found it by the end of that time, they were lead to the platform. They remained on the platform for 30 seconds before being removed to their cages to rest. The probe trials were designed to assess the animal's memory or spatial bias for the platform location [54]. The naïve probe trial was used to determine if there were any pre-existing biases and to be able to show improvement in probe trial performance. The platform was not present during the probe trials and the animal was allowed to search for 30 seconds and then was removed to a cage.

#### Cued trials

On a separate day, after or prior to place learning, depending on the group, the cued trials were run. The platform was submerged, but was marked by an 8-inch tall support with a flag. For each animal's trial, the platform position was changed to a different quadrant. The platform locations for the cued trials were as follows: 1 – South (close to the wall), 2 – Center of tank, 3 – NE, 4 – North (half of the distance between a quadrant position and the wall), 5 – SE and 6 – NW. For each trial, the animal was placed into the tank, facing the wall at one of the entry points, and was allowed to search for the platform for 60 seconds. If the animal did not find the platform by the end of that time, they were lead to it. Each animal was tested at one platform position, then the platform was moved. This continued until all 6 positions were used. Cued trials were designed to test visual acuity, physical ability, and motivation for the task.

#### Analysis

Cumulative proximity was used to measure performance in the place and cued trials. Cumulative proximity was calculated by the Poly-Track system according to the method of Gallagher et al. [54], including correcting for start position. Briefly, the animal's distance from the platform, or proximity measure, was measured by the computer every frame for the duration of the animal's swim. These proximity measures were then added together to give a cumulative proximity. The Poly-Track software also corrected for start position by calculating the time to directly swim to the platform, based on swim speed and starting point and removed the data for this time period from the initial part of the record prior to calculating cumulative proximity. Average proximity to the platform was used to assess performance in the probe trials [54]. The data was collected similar to the cumulative proximity measure, but after correcting for starting point, the proximity measures were averaged [54]. Learning index scores were calculated from the probe trial data according to Gallagher et al., [54]. The mean average proximity measurements for the young mice in the naïve probe trial (probe trial 0) were divided by the mean measurements for the young mice in each separate probe trial in order to obtain a multiplier for each probe trial. The multipliers obtained were as follows: 1.00, 1.38 and 1.69 for probe trials 0–2, respectively. For each mouse, the average proximity scores for each trial were multiplied by the respective multipliers for each trial and the products were summed to obtain a learning index score for that mouse. For both cumulative and average proximity and learning index scores, higher values represented poorer learning ability and lower values indicated better learning performance. Proximity measures were used to assess performance in these studies because they are less influenced by swim speed differences than more traditional measures such as latency to reach the platform [17,54]. The proximity measures are also more sensitive to some of the alternative strategies that animals can use to find the platform that may not involve place learning [54]. The learning index score provides similar information to traditional measurements of time spent in the correct quadrant, but has the added advantage of providing a single value that can represent the spatial bias in multiple probe trials and also reflect the learning curve by being weighted to reward those animals who acquire the task faster [54].

### Tissue dissection

Ten days after behavioral testing, the mice were euthanized by exposure to CO_2_, followed by decapitation. The brains were removed, frozen on dry ice, and stored in a -70°C freezer. The mice that underwent reference testing prior to cued testing were used for the assessment of NMDA receptor subunit expression. This was similar to the protocol that we have used previously with longer-term reference memory tasks [18,19] and there was some concern that the cued trials may have contributed to some spatial learning in the mice tested in the cued trials first. One half of the brain was warmed to -20°C and placed in a plastic brain mold (Braintree Scientific, Braintree, MA) on ice and cut in the coronal plane. The rostral 3 mm of cortex were dissected and used for semi-quantitation of the protein expression of the ζ1, ε1, and ε2 subunits of the NMDA receptor and syntaxin by Western blotting. These prefrontal/frontal dissections included orbital, limbic, insular, cingulate, primary and association motor and sensory cortices [84]. Olfactory bulb, caudate nucleus and brainstem were dissected away from the cortex and discarded. The hippocampus was isolated, removed, and used to analyze the same proteins described above. The remaining caudal cortex (including parietal, occipital and temporal cortices) from the 3-month-old mice was used to produce standard curves for protein analysis. Brains were randomly assigned to an assay group, which consisted of multiple representatives of each age. The brains within an assay group were processed and assayed at the same time.

### Crude synaptosome preparation

Crude synaptosomes (P2 fraction) from the prefrontal/frontal cortex and hippocampus were prepared separately as described by Dunah and Standaert [53] with some modifications. The caudal cortices from the 3 month old mice were processed along with the samples of prefrontal/frontal cortex and hippocampus and then combined prior to the protein assay for use as cortical standards. Dissected brain regions were placed in Dounce homogenizers containing ice-cold HEPES buffer (10 mM HEPES, 1 mM EDTA, 10% sucrose, pH 7.4) plus a protease inhibitor cocktail (2 μl/ml buffer; Sigma, St. Louis, MO) and homogenized by hand. The homogenates were centrifuged at 1000 × *g *for 8 minutes in an Eppendorf microcentrifuge (Brinkmann, Westbury, NY). The pellet was discarded. The supernatent was centrifuged at 9500 × *g *for 15 minutes. The supernatent was discarded and the pellet (crude synaptosomal fraction (P2)) was reconstituted in ice-cold HEPES buffer plus protease inhibitors as described above. The P2 fractions were tested for protein concentration using the bicinchoninic acid (BCA; Pierce, Rockford, IL) assay, diluted in buffer 2 to 5 mg/ml protein, aliquoted and stored at -70°C.

### Western blots

Western blotting was performed as described by Dunah and Standaert [53]. Aliquots of the membrane preparations were thawed, diluted in 2% sodium dodecyl sulfate (SDS) and 50 mM dithiothreitol, and boiled for 5 minutes. Five different amounts of protein (1.5 – 12 μg) from the caudal cortex were loaded on each gel as standards along with 1.5–3 μg of protein from the prefrontal/frontal region or hippocampus from each animal in one assay group. SDS-PAGE (7.5%) gels were run in triplicate and transferred to Sequi-Blot PVDF membrane [85]. The positions of representatives for each age group were alternated across the gel. Separate gels were used for the ε1 and ε2 subunits because they are the same molecular weights [45,46]. Strips of each gel containing the appropriate molecular weight range for each protein of interest were cut and blotted separately. The membranes were blocked in 5% Carnation nonfat dry milk in Tris-buffered saline (TBS; 20 mM Tris-HCl, 140 mM NaCl, pH 7.2) with 0.05% Tween-20 (TBS-T) for 1 hour at room temperature and were incubated overnight at 4°C in blocking buffer containing primary antibodies. The antibodies to identify the ζ1, ε1, and ε2 subunits of the NMDA receptor were purchased from Zymed (So. San Francisco, CA) and the syntaxin antibody was obtained from Sigma (St. Louis, MO). The membranes were rinsed 4 times for a total of 20 minutes in TBS-T, incubated in horseradish peroxidase-conjugated secondary antibody diluted in blocking buffer for 1 hour at room temperature, and rinsed 5 times for 35 minutes total in TBS-T. The membranes were then incubated in ECL SuperSignal West Pico solution (Pierce, Rockford, IL) and the bands were visualized by opposing the blots to ECL Hyperfilm.

Film images were scanned into a G4 Macintosh computer with the use of a PowerLook II scanner (UMAX, Taiwan). The integrated densities of the bands were analyzed with the use of the Gel Plotting Macro in NIH Image software. Standard curves were obtained with the use of Prism software (GraphPad Software, San Diego, CA) using a non-linear regression fit (sigmoidal dose-response (variable slope option) [86]. Sample bands were analyzed, interpolated from the standard curve and expressed as μg cortical protein equivalents. Sample bands that had densities within the saturated portion of the standard curve were not used.

### Data analysis

Statistical analysis for behavior was performed by repeated measures ANOVA (age × trial), followed by Fisher's protected LSD where applicable. The age analyses within specific trials were part of the original experimental design. Age-related differences in protein for the crude synaptosomes were analyzed by one-way ANOVA (age), followed by Fisher's protected LSD where indicated. Correlations between behavioral measurements (overall place performance and learning index score) were examined using Pearson correlation coefficients. Values of p ≤ .05 were considered significant. All statistical analyses were performed with the use of Statview software (SAS Institute, Inc., Cary, N.C.).

## Abbreviations

ANOVA analysis of variance

BCA bicinchoninic acid

CCD charged coupled device

CPP [(±)-2-carboxypiperazin-4-yl] propyl-1-phosphonic acid

ε1 (NR2A) NMDA receptor subunit epsilon1 (2A)

ε2 (NR2B) NMDA receptor subunit epsilon2 (2B)

ECL enhanced chemiluminescence

EDTA ethylenediaminetetraacetic acid

MK801 dizocilpine

mRNA messenger ribonucleic acid

NE northeast quadrant

NMDA N-methyl-D-aspartate

NW northwest quadrant

P2 crude synaptosome fraction

SDS sodium dodecyl sulfate

SDS-PAGE sodium dodecyl sulfate – polyacrylamide gel electrophoresis

SE southeast quadrant

TBS Tris-buffered saline

TBS-T Tris-buffered saline – Tween 20

ζ1 (NR1) NMDA receptor subunit zeta1 (1)

## Authors' contributions

KM conceived of the study, performed the behavioral tasks, supervised the protein work and drafted the manuscript. BS contributed to the design and discussion and performed the behavioral tasks. XZ and RH performed the protein studies. All authors read and approved the final manuscript.
